# Multi‐decadal environmental change in the Barents Sea recorded by seal teeth

**DOI:** 10.1111/gcb.16138

**Published:** 2022-03-01

**Authors:** Camille de la Vega, Pearse J. Buchanan, Alessandro Tagliabue, Joanne E. Hopkins, Rachel M. Jeffreys, Anne Kirstine Frie, Martin Biuw, Joanna Kershaw, James Grecian, Louisa Norman, Sophie Smout, Tore Haug, Claire Mahaffey

**Affiliations:** ^1^ 4591 School of Environmental Sciences University of Liverpool Liverpool UK; ^2^ National Oceanography Centre Liverpool UK; ^3^ Institute of Marine Research Fram Centre Tromsø Norway; ^4^ 7486 Sea Mammal Research Unit Scottish Oceans Institute University of St Andrews St Andrews UK; ^5^ Present address: Leibniz Institute for Baltic Sea Research, Warnemünde Rostock 18119 Germany

**Keywords:** Arctic, Atlantification, atmospheric nitrogen deposition, harp seal, stable nitrogen isotopes

## Abstract

Multiple environmental forcings, such as warming and changes in ocean circulation and nutrient supply, are affecting the base of Arctic marine ecosystems, with cascading effects on the entire food web through bottom‐up control. Stable nitrogen isotopes (δ^15^N) can be used to detect and unravel the impact of these forcings on this unique ecosystem, if the many processes that affect the δ^15^N values are constrained. Combining unique 60‐year records from compound specific δ^15^N biomarkers on harp seal teeth alongside state‐of‐the‐art ocean modelling, we observed a significant decline in the δ^15^N values at the base of the Barents Sea food web from 1951 to 2012. This strong and persistent decadal trend emerges due to the combination of anthropogenic atmospheric nitrogen deposition in the Atlantic, increased northward transport of Atlantic water through Arctic gateways and local feedbacks from increasing Arctic primary production. Our results suggest that the Arctic ecosystem has been responding to anthropogenically induced local and remote drivers, linked to changing ocean biology, chemistry and physics, for at least 60 years. Accounting for these trends in δ^15^N values at the base of the food web is essential to accurately detect ecosystem restructuring in this rapidly changing environment.

## Graphical abstract

Bottom‐up relative to top‐down control has been predicted to increase in Arctic food webs. Detecting changes and determining their drivers at the base of the Arctic food web is therefore crucial for forecasting food web dynamics. Combining stable nitrogen isotopes (δ^15^N) of phenylalanine from archived teeth of harp seals, and results from an ocean‐biogeochemical model, we show a 60‐year decline of δ^15^N values at the base of the Barents Sea food web, driven by anthropogenic atmospheric nitrogen deposition in the Atlantic, increased northward transport of Atlantic water through Arctic gateways and local feedbacks from increasing Arctic primary production.

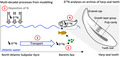

## 1 INTRODUCTION

The Arctic Ocean is changing rapidly as a direct result of anthropogenic activities (Meredith et al., 2019). As the physical and biogeochemical environment responds, changes in local primary production (Dalpadado et al., 2020; Lewis et al., 2020) and ecosystem dynamics (Kortsch et al., 2015) are expected. Continued ocean warming has been predicted to increase the resource supply (termed bottom‐up) relative to consumer pressure (termed top‐down) control in food webs located in cold high latitude seas (Boyce et al., 2015; Dalpadado et al., 2020; Johannesen et al., 2012), with productivity at higher trophic levels being increasingly constrained by variability at the base of the food web (Ware & Thomson, 2005). This means that detecting changes at the base of Arctic food webs and determining their drivers is crucial for forecasting and managing modifications that cascade though the entire food web. However, monitoring changes in polar marine environments over multiple decades is logistically challenging and the lack of long‐term observations, both locally and at pan‐Arctic scales, hampers our understanding of how anthropogenic activities affect the base of Arctic food webs.

The stable isotopes of nitrogen (expressed as δ^15^N in ‰, where δ^15^N = [(^15^N/^14^N)_sample_/(^15^N/^14^N)_air_ – 1] × 1000) are useful for detecting environmental change. In pelagic marine systems, phytoplankton form the base of the food web and rely on nitrate as an essential source of nitrogen for biosynthesis. In assimilating nitrate into their cells, phytoplankton integrate the isotopic signature of nitrate (δ^15^N_NO3_) into their organic matter (δ^15^N_POM_). δ^15^N_NO3_ values are themselves highly sensitive to environmental change, reflecting shifts in the presence of different water masses (Tuerena et al., 2015), inputs of terrestrially derived nitrogen (Altieri et al., 2016), and variations in the rate of in situ biogeochemical processes (Cochran et al., 2019), namely primary production, remineralization, biological nitrogen fixation and denitrification in deoxygenated settings (Sigman & Fripiat, 2019). Nitrate is supplied to the Arctic Ocean by Atlantic water entering through the Barents Sea, and by Pacific water crossing the Bering Strait (Torres‐Valdes et al., 2013). Within the Arctic, primary production and denitrification elevate δ^15^N_NO3_ values, terrestrially derived nitrogen inputs decrease δ^15^N_NO3_ values, and shifts in circulation can increase or decrease values. Atlantic waters, for instance, have lower values than both Pacific inflows and the water masses present in the high Arctic (Knapp et al., 2008; Marconi et al., 2015; Tuerena et al., 2021; de la Vega et al., 2020). Bottom‐up changes involving the circulation, sources of nitrogen and/or biogeochemical processes, such as primary production, are thus recorded by δ^15^N_NO3_ values and assimilated into phytoplankton organic matter (δ^15^N_POM_).

The Arctic and especially the Barents Sea, which is the main gateway between the Arctic and adjacent Atlantic Ocean, are experiencing simultaneous changes in water mass circulation, the rate of Atlantic and Pacific inflows (Polyakov et al., 2020; Woodgate, 2018), nitrogen sources and in situ biogeochemical processes. First, the overall volume (Oziel et al., 2016) and speed (Oziel et al., 2020) of Atlantic water flowing northward has increased in the last decades, bringing more low δ^15^N_NO3_ values into the Barents Sea. Second, Atlantic water entering the Barents Sea is composed of North Atlantic subpolar and subtropical origin waters (Hátún et al., 2017), and the proportion of subtropical water entering the Barents Sea, which has lower δ^15^N_NO3_ values than subpolar water (Knapp et al., 2008; Marconi et al., 2015), has increased over the past three decades (Hátún et al., 2017). Third, since the beginning of the twentieth century, increased use of industrial nitrogen fertilizers as well as the burning of fossil fuels has more than doubled the atmospheric deposition of ^15^N‐depleted nitrogen to the Atlantic Ocean (Duce et al., 2008; Jickells et al., 2017; Yang & Gruber, 2016). Finally, increasing light and nutrient utilization has increased primary production within the Barents Sea (Lewis et al., 2020; Lind et al., 2018), which in contrast to the previous changes may elevate δ^15^N_NO3_ values. Overall, the Barents Sea is transitioning from a cold, salinity‐stratified shelf sea system into a warmer, less‐stratified Atlantic‐dominated regime (Lind et al., 2018). This transition should be registered by detectable shifts in δ^15^N_NO3_ and δ^15^N_POM_ values at the base of the food web, but due to logistical limitations of on‐site sampling this polar environment, long‐term data series of δ^15^N_NO3_ or δ^15^N_POM_ measurements are lacking to evaluate if and when changes are occurring.

Biomarker analyses of archives of predator tissues offer a unique solution to overcome these limited sampling opportunities. The δ^15^N values of phenylalanine (δ^15^N_Phe_) in predator tissue reflects the δ^15^N values at the base of the food web, herein considered to be that of marine phytoplankton organic matter (δ^15^N_POM_), and provides insight into the oceanographic and biogeochemical changes occurring in their habitat (de la Vega et al., 2020). This is because phenylalanine exhibits minimal fractionation during trophic transfer (McMahon & McCarthy, 2016), and thus δ^15^N_NO3_ variations that are integrated into δ^15^N_POM_ values are conserved within the δ^15^N_Phe_ values of subsequent consumers. Measurement of δ^15^N_Phe_ values in the inert tissue of predators, such as the dentine layers in marine mammal teeth, can reconstruct chronological records of δ^15^N values at the base of the food web (e.g. δ^15^N_POM_) over periods not possible through direct observation (Feddern et al., 2021; Hobson & Sease, 1998; Newsome et al., 2007).

Here, we report a 60‐year decline of δ^15^N values at the base of the Barents Sea food web using δ^15^N_Phe_ values from archived teeth of an Arctic predator, the harp seal (*Pagophilus groenlandicus*) and explore the environmental drivers of this decline, including transport of Atlantic water, atmospheric nitrogen deposition and primary production, using an ocean‐biogeochemical model (Aumont et al., 2015) equipped with nitrogen isotopic tracers (δ^15^N_NO3_ and δ^15^N_POM_; Buchanan et al. (2021)). Harp seals are an abundant ice‐associated generalist top predator of the Barents Sea (Lindstrøm et al., 2013; Nilssen et al., 1995; Øigård et al., 2013). As their migration range is restricted within the Barents Sea (Haug et al., 1994; Nordøy et al., 2008), the δ^15^N_Phe_ values of harp seals are good indicators of environmental changes occurring at the base of the food web in this region. This record extends beyond the time period accessible from satellites and direct oceanographic observations. Combined with results from our ocean‐biogeochemical model, we show that anthropogenically forced environmental changes occurred in the Barents Sea beginning as early as the 1950s.

## 2 MATERIALS AND METHODS

We measured the δ^15^N_Phe_ values of harp seal teeth from the Barents Sea from 1951 to 2012 (de la Vega et al., 2021). In addition, we used a fully coupled, three‐dimensional ocean‐biogeochemical model to produce historical conditions from 1970 to 2019. Temporal trends in δ^15^N_NO3_ and δ^15^N_POM_ values were extracted at the Barents Sea Opening and within the Barents Sea region (Buchanan et al., 2022). This region was constrained by harp seal telemetry. In addition to these isotopic trends, we also assessed how the transport of Atlantic Water and nitrate across the Barents Sea Opening changed and how nitrate concentrations and vertically integrated net primary production changed within the Barents Sea (Buchanan et al., 2022). Separate experiments with and without the anthropogenic increase in atmospheric nitrogen deposition were conducted to quantify its effect on these properties.

### 2.1 Harp seal sampling for stable nitrogen isotope analyses

**FIGURE 1:**
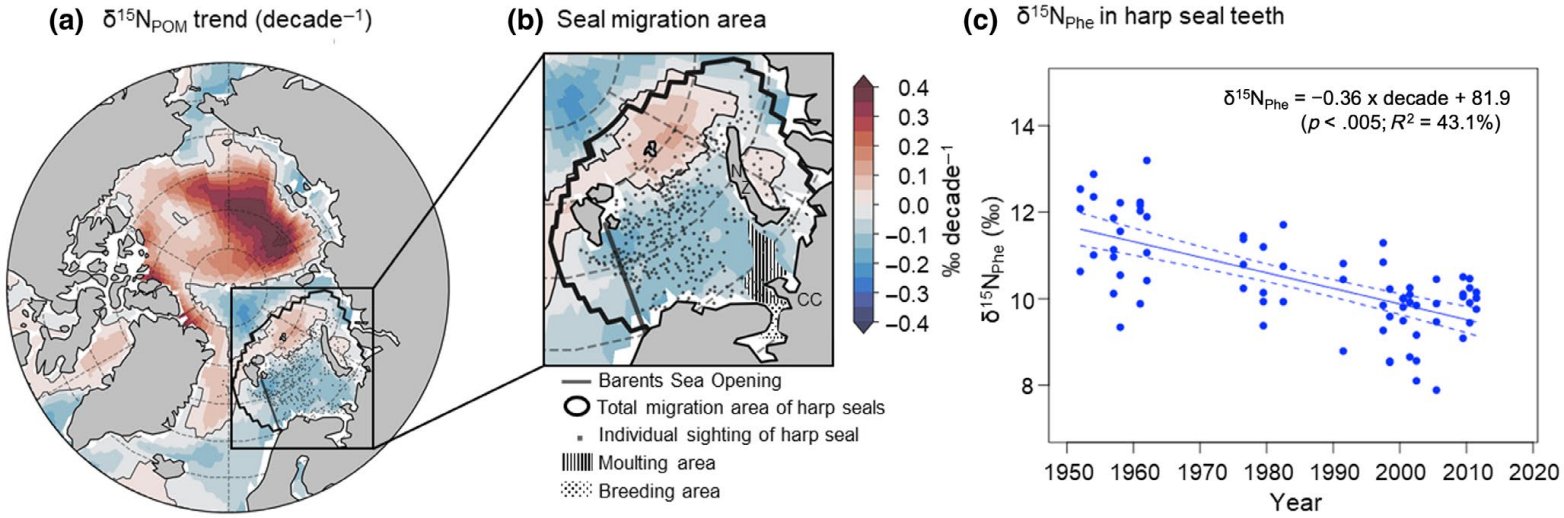
Decadal trends in δ^15^N at the base of the Barents Sea food web; Average linear trend in δ^15^N values of particulate organic matter (δ^15^N_POM_) per decade in the Arctic Ocean (a) and the Barents Sea (b) over simulation years 1970–2019 including atmospheric N_r_ deposition; Total migration area of harp seals (solid line) and sites of Norwegian seals’ presence (SI), NZ = Nova Zemlja, CC = Cap Canin; the dark grey line in panels (a) and (b) is the transect across the Barents Sea Opening used in Figure 2; (c) Decadal trends in δ^15^N_Phe_ in harp seal teeth from the Barents Sea; Each data point represents the δ^15^N_Phe_ value integrated in two growth layer groups combined for analyses, representing a 2‐year period, which corresponds to the second and third year of life of each seal individual

Teeth of harp seals (*n* = 72) were taken from archives of the Norwegian Institute for Marine Research (IMR). All seals were hunted as part of the Norwegian commercial harvest, and the seals were sampled as part of IMR monitoring. All individuals were sampled around Cap Canin and west of the Novaja Zemlja pack ice during the Norwegian commercial seal hunt (Figure 1a,b).

### 2.2 Harp seal sample preparation

We analysed the δ^15^N_Phe_ values in dentine of harp seals from two annual growth layer groups (GLGs) representing the second and third year of life, as described in detail in (Kershaw et al., 2021; Data S1, Figure S1). In brief, the roots of the teeth were sectioned along two planes, transverse and sagittal using a precision low speed diamond saw (Buehler, Isomet^TM^). The transverse sections were used to determine the different growth layer groups (GLG) by the structure, width and opacity of individual layers (Bowen et al., 1983). Each GLG corresponds to 1 year of life of the individual. A 700 µm sagittal section was cut as close as possible to the central plane of the tooth and de‐mineralized with 0.25 M HCl for between 12 and 24 h. Once softened, any remaining gum tissue and cementum was cut away from the outer edge of the tooth. The pure dentine samples representing the individual GLGs for the second (GLG2) and third (GLG3) years of life were combined, freeze‐dried and stored in plastic vials until stable isotope analysis. GLG2 and GLG3 were combined for each individual to have enough sample mass for analyses. GLGs covered years from 1951 to 1952 and from 2011 to 2012 (Data S1, Figure S1).

### 2.3 Stable isotope analyses

Nitrogen isotope analyses were carried out at the Liverpool Isotopes for Environmental Research laboratory, University of Liverpool, and reported in standard δ‐notation (‰) relative to atmospheric N_2_. For compound specific δ^15^N analyses on the source amino acid phenylalanine (δ^15^N_Phe_), ~15 mg of dentine was hydrolyzed in 1‐ml reaction vessels (200 µl, 6 M HCl, 100°C for 22 h). L‐Norleucine (Sigma‐Aldrich) was added to each sample as an internal standard (80 µl of 5 mg ml^−1^). On cooling, the samples were transferred into a nanosep centrifugal device (45 µm nylon filters) and centrifuged (10,000 rpm for 1 min). Samples were then transferred into clean micro‐reaction vessels and lipids were extracted by addition of n‐hexane:DCM (3:2 v/v, 0.5 ml). Each sample was shaken by hand for 10 s to mix the hydrolysate and solvents. Then, organic solvents were removed. This was repeated three times. Hydrolysates were blown down under N_2_ for 2 min to ensure that all organic solvents were removed and were frozen at −80°C prior to lyophilization. The amino acids were propylated in 0.25 ml of acidified isopropanol solution (prepared by addition of acetyl chloride to anhydrous isopropanol (1:4 v/v) in an ice bath) at 100°C for 1 h. The reaction was quenched in a freezer and reagents were evaporated under a gentle stream of N_2_. DCM was added (3 × 0.25 ml) and evaporated to remove excess reagents. Amino acid methyl esters were then treated with 1 ml of a mixture of acetone:triethylamine:acetic anhydride (5:2:1, v/v), which was added to each sample, and heated at 60°C for 10 min. Following acetylation, the reagents were evaporated under a gentle stream of N_2_ and were dissolved in 2 ml of ethyl acetate, to which 1 ml of saturated NaCl solution was added. Phase separation was enabled via mixing and the organic phase was collected. Separation was repeated three times with addition of 2 ml of ethyl acetate. Residual water was removed from the combined organic phases by passing through a Pasteur pipette plugged with glass wool and filled with MgSO_4_. Finally, samples were evaporated under N_2_, and the derivatized amino acids were dissolved in DCM and stored at −20°C until analysis.

δ^15^N_AA_ values were determined using a Trace Ultra gas chromatograph (GC) coupled to a Delta V Advantage IRMS with a ConFlo IV interface (Cu/Ni combustion reactor held at 1000°C, Thermo Fisher). A liquid nitrogen trap was added after the reduction oven to remove CO_2_ from the sample stream. The separation of amino acids was achieved using an HP Innowax capillary column (30 m × 0.25 mm i.d. × 0.5 µm film thickness, Agilent). Each sample was introduced to the column using a split/splitless injector set at 260°C. The GC was programmed as follows: held at 50°C for 2 min, 10°C min^−1^ to 180°C and 6°C min^−1^ to 260°C, and held for 16.7 min. The carrier gas was ultra‐high‐purity helium (flow 1.4 ml min^−1^). The ion intensities of *m*/*z* 28, 29 and 30 were monitored, and the δ^15^N values of each amino acid peak were automatically computed (Isodat version 3.0; Themo fisher) by comparison with a standard reference N_2_ gas, which was repeatedly measured (×4) at the beginning and the end of each sample analysis.

All results were reported in per mil (‰) relative to N_2_. Each sample was analyzed in duplicate, and a triplicate measurement was made if the mean δ^15^N_Phe_ values fell outside the expected measurement error (<1.0 ‰). Precision and accuracy were determined using a mixed amino acid standard prepared from seven amino acids (alanine, valine, leucine, aspartic acid, glutamic acid, glycine and phenylalanine) with known δ^15^N values (University of Indiana, USA and SI Science Japan). The mixed standard was analysed every 4 injections. The mean precisions and accuracies were ±0.9 ‰ and ±0.7 ‰ (1σ, *n* = 29), respectively. The precisions and accuracy of δ^15^N values of phenylalanine were ±0.5 ‰ and ±1.1 ‰ (1σ, *n* = 29), respectively. Raw δ^15^N_AA_ sample values were corrected following the methods of (McCarthy et al., 2013). This method takes into consideration the response of individual amino acids to the stationary phase of the column and is based on the offset between the measured δ^15^N_AA_ values in the nearest mixed standard and their known δ^15^N_AA_ values (Equation 1).

(1) $$\begin{array}{c} \delta^{15}{\text{N‐Sample}}_{\text{reported}}=\text{Avg}\delta^{15}{\text{N‐Sample}}_{\text{measured}}‐\left(\delta^{15}{\text{N‐Standard}}_{\text{measured}}‐\delta^{15}N_{\text{known}}\right), \end{array}$$

where Avgδ^15^N‐Sample_measured_ is the average δ^15^N for an amino acid in a sample (*n* = 2), δ^15^N‐Standard_measured_ is the δ^15^N for the AA in the nearest mixed standard and δ^15^N_known_ is the known value for the same standard.

### 2.4 Statistical analyses of harp seal stable isotope data

Statistical analyses were performed in R version 3.5.1 (R Core Team, 2018). To analyse temporal variation in δ^15^N_Phe_ values in harp seals, a linear model was fitted with δ^15^N_Phe_ values as a function of year. We used the Gaussian family with no transformation of the data, assuming that measurement errors were normally distributed. Model fit was checked by residual analyses with visual inspection of residuals versus fitted values (Data S1, Figure S2a) to verify homogeneity, residuals *versus* explanatory variable (i.e. year; Data S1, Figure S2b) to check independence and quantile‐quantile plot of the residuals for normality (Data S1, Figure S2c) (Zuur et al., 2009). *p*‐values, *R* ^2^, *F*‐statistics and degrees of freedom are reported (Data S1, Table S1).

### 2.5 Harp seal migration pattern, telemetry

To constrain the area annually integrated by harp seals, we used telemetry data from 15 satellite relay data loggers fitted to individuals from the Barents Sea/White Sea population in 1995 and 1996 (Nordøy et al., 2008). The region of interest for harp seal migration was estimated as the 95% contour of the bivariate normal kernel utilization distribution of telemetered locations, using a smoothing parameter of 250 km (Calenge, 2006). In addition, individual sightings of harp seals archived by Norway and Russia from 1965 to 1993 were used to ground‐truth our computed distribution based on the telemetry data (Haug et al., 1994).

### 2.6 Model simulations

The global ocean‐biogeochemical model (Pelagic Interactions Scheme for Carbon and Ecosystem Studies version 2 (PISCES‐v2; Aumont et al., 2015) was used with explicit consideration of δ^15^N cycling (Buchanan et al., 2021). This biogeochemical model was coupled to the Nucleus for European Modelling of the Ocean version 4.0 (NEMO‐v4.0) general ocean circulation model and the Sea Ice modelling Integrated Initiative (SI^3^) sea ice model to simulate a large suite of oceanographic and biogeochemical conditions. The ecosystem component of PISCES‐v2 includes two phytoplankton types (nanophytoplankton and diatoms) and two zooplankton types (microzooplankton and mesozooplankton). Biogeochemical tracers include five nutrients (nitrate, ammonium, iron, phosphate, silicate), oxygen, the full carbon cycle, dissolved organic matter, chlorophyll, iron‐complexing ligands and large and small particulate organic matter. Nitrogen cycling involves internal cycling by the ecosystem component, loss processes of denitrification, anammox and burial in the sediments, as well as sources of nitrogen from biological nitrogen fixation, rivers and atmospheric deposition. Horizontal model resolution varied between ~0.5° at the equator, 1° poleward of 50°N and 50°S, and 2° in the subtropics, whereas vertical resolution varied between 10 and 500 m thickness over 31 levels, with 10 levels in the upper 100 m.

The model was used to produce historical conditions from 1970 to 2019. Historical simulations followed the protocols of the Ocean Modelling Inter‐comparison Project (Orr et al., 2017), where the model was forced by the Japanese atmospheric reanalysis over the years 1958 to 2019 (Tsujino et al., 2018). Six repeat cycles as recommended (Tsujino et al., 2020) of this 62‐year forcing (372 years) were made beginning on 1 January 1648, ending on 31st December 2019. Tracer fields (e.g. nitrate) were initialized on 1 January 1648 using the solution of a 5000 year spin‐up simulation conducted under preindustrial conditions. Thereafter, the beginning of each cycle was initialized with the end of the previous cycle. Only output in the final cycle was used in analysis, and the first 12 years (1958–1969) were not included due to unavoidable initialization with the final year (2019) of the previous cycle. A model‐data assessment is detailed in Data S3.

Atmospheric deposition of nitrogen evolved over the simulation according to historical measurements and reconstructions. Prior to year 1851, atmospheric deposition was held at its preindustrial rate of 11 teragrams year^−1^. Onwards from 1851, atmospheric deposition was increased using fields of (Hauglustaine et al., 2014) according to the increase in anthropogenic activities. Linear interpolation was used to estimate years in‐between those estimated by Hauglustaine et al. (2014), being 1850, 2000 and 2030. However, to represent the rapid increase in anthropogenic activities contributing to increased nitrogen deposition since 1950 (Galloway et al., 2004), 60% of the increase between 1850 and 2000 occurred from 1950 onwards. The simulation without the anthropogenic increase in nitrogen deposition maintained a constant preindustrial rate.

### 2.7 Extraction of data from the model

Part of our analysis focussed on the transport of water, nitrate and δ^15^N_NO3_ values across the Barents Sea Opening (20° East between Norway and Svalbard). Volume transport in Sverdrups (Sv = 10^6^ m^3^ s^−1^) of Atlantic Water (potential temperature >2°C; salinity >34.5 psu) was calculated by integrating the zonal velocities in both depth and latitude. We then calculated the contribution from velocity changes and volume changes to net transport. To do this, we divided the transport rate by the area of Atlantic Water, and then multiplied this by the average area of Atlantic Water over the full simulation. This provided the volume‐normalized transport rate. The difference between the multi‐decadal linear trend of transport and volume‐normalized transport was the contribution of volume changes of Atlantic Water (Data S2, Figure S1). The transport of nitrate across the Barents Sea Opening was calculated by multiplying nitrate concentrations by the zonal velocities and then integrating in both depth and latitude. Average nitrate and δ^15^N_NO3_ values carried across the Barents Sea Opening in Atlantic Water were calculated by averaging these properties only within Atlantic Water.

The other part of our model analysis focused on changes in properties within the area sampled by harp seals in the Barents Sea. This region agreed with point location data from Norwegian harp seal sightings and sampling (Figure 1a,b), thus providing confidence that our data extraction from this region was representative of the conditions that harp seals experienced. Isotopic values of nitrate (δ^15^N_NO3_) and particulate organic matter (δ^15^N_POM_) averaged over the upper 100 m (first 10 levels) were extracted at each grid cell for each simulated year within the harp seal migration area. Net primary production in the Barents Sea was calculated by vertically and horizontally integrating across those grid cells within the same area. Average nitrate concentrations were calculated by averaging within the upper 100 m and within the harp seal migration area (Data S2, Figure S2).

## 3 RESULTS

### 3.1 Temporal trend in δ^15^N_Phe_ values in harp seal teeth in the Barents Sea

δ^15^N values and rates of change are expressed as mean ± standard deviation. The total area occupied by harp seals was acquired from independent harp seal sightings from 1965 to 1993 and was restricted to the Barents Sea (Figure 1a,b). Since the 1950s, the δ^15^N_Phe_ values in harp seal teeth from the Barents Sea showed a clear trend of −0.36 ± 0.01 ‰ decade^−1^ (linear model: *p* < .01, *R* ^2^ = 43.1%, *F* = 54.8, *n* = 72, Data S1, Figure S2 and Table S1), declining from 11.7 ± 1.0 ‰ in 1951–1952 to 10.0 ± 0.2 ‰ in 2011–2012 (Figure 1c).

### 3.2 Simulated trends

**FIGURE 2:**
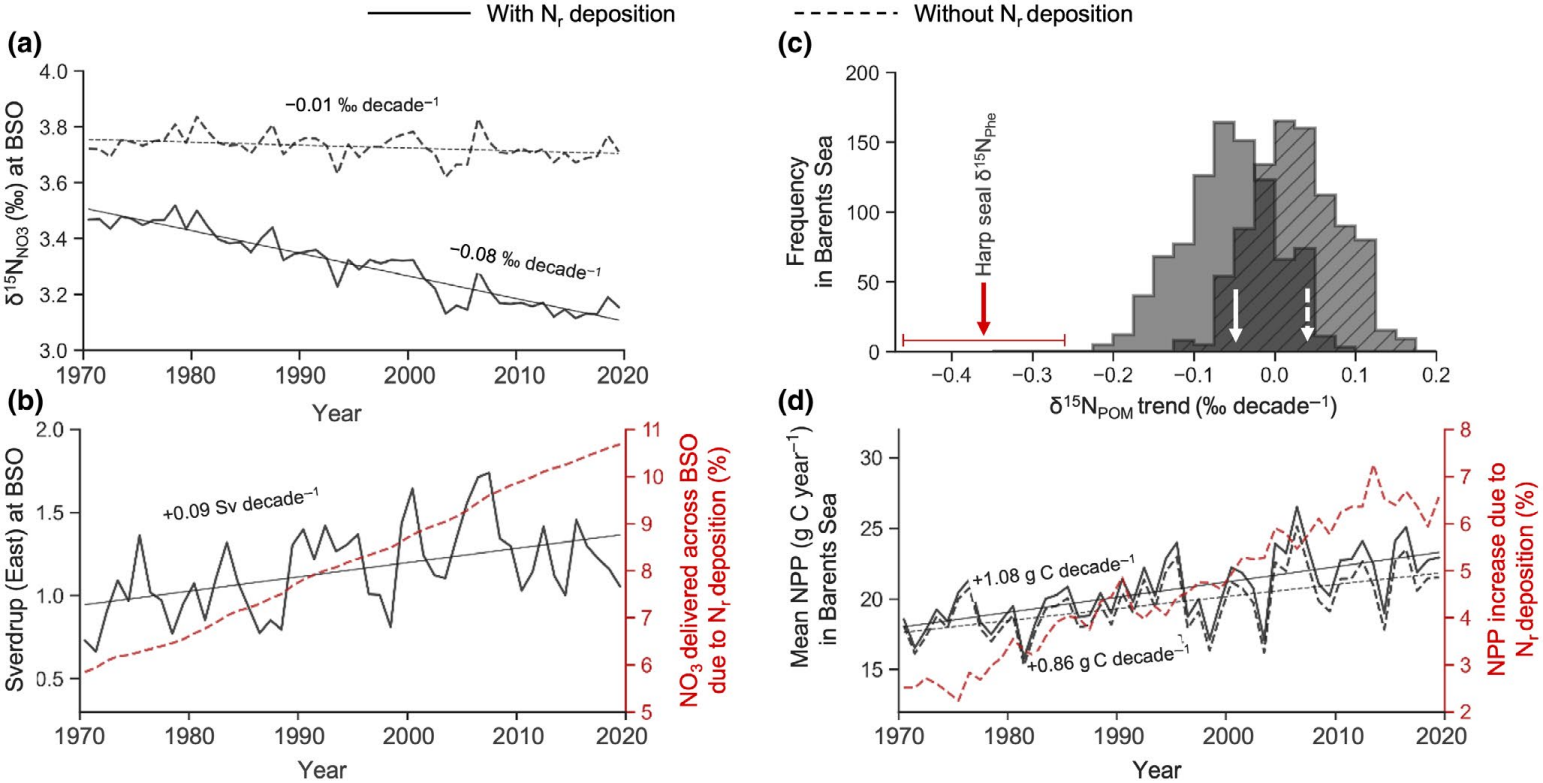
Simulated time series of properties at the Barents Sea Opening (BSO) and changes to the isoscape and primary production within the Barents Sea. Simulations with (solid) and without (dashed) atmospheric reactive nitrogen (N_r_) deposition are shown. (a) Mean δ^15^N_NO3_ values of Atlantic Water crossing the BSO and entering the Barents Sea at 20°E; (b) Integrated volume transport of Atlantic Water (black) in Sverdrup (Sv = 10^6^ m^3^ s^−1^) and the percent increase in cumulative nitrate (NO_3_) delivered through the BSO due to anthropogenic N_r_ deposition (red); (c) decadal trends of δ^15^N_POM_ values averaged over the upper 100 m extracted each model grid cell within the total migration area of harp seals shown in Figure 1, and mean decadal trend of δ^15^N_POM_ values within the total migration area of harp seals with (plain white arrow) and without (dashed white arrow) increased anthropogenic atmospheric N_r_ deposition, and range (red horizontal line) and mean (red arrow) decadal trend of δ^15^N_Phe_ values in harp seal teeth; (d) mean vertically integrated net primary production (NPP) within the total migration area of harp seals shown in Figure 1, and the percent increase in NPP due to anthropogenic N_r_ deposition (red). Atlantic Water is defined as water >2°C and saltier than 34.5 psu

At the Barents Sea Opening (20°E between Norway and Svalbard), our model predicted that the average δ^15^N_NO3_ values of inflowing Atlantic Water (potential temperature >2°C; salinity >34.5 psu) decreased by −0.08 ± 0.00 ‰ decade^−1^ from 1970 to 2019 (Figure 2a). In a parallel simulation without the anthropogenic increase in nitrogen deposition, the rate of the decline in δ^15^N_NO3_ values was much reduced at −0.01 ± 0.00 ‰ decade^−1^. Meanwhile, the eastward volume transport of Atlantic Water across the Barents Sea Opening increased by +0.09 ± 0.02 Sv decade^−1^ (Figure 2b), with two thirds of this trend driven by a volumetric increase of Atlantic Water and one third by an increase in eastward velocities (Data S2, Figure S1). The flux of nitrate in Atlantic Water entering the Barents Sea also increased, consistent with the greater volumetric inflow. However, this flux of nitrate was augmented by anthropogenic nitrogen deposition (Figure 2b). Thus, between 1970 and 2019, roughly 5% more nitrate with a lower δ^15^N_NO3_ signature entered the Barents Sea when anthropogenic nitrogen deposition was accounted for.

In the Barents Sea, the trend in δ^15^N_POM_ values within the harp seal migration area ranged from −0.22 to +0.08‰ decade^−1^ and was on average −0.05 ± 0.01‰ decade^−1^ with the anthropogenic increase in nitrogen deposition included (Figures 1b and 2c). Without the increase in anthropogenic deposition, the trends in δ^15^N_POM_ values ranged from −0.11 to +0.15‰ decade^−1^ and were generally positive (+0.03 ± 0.01‰ decade^−1^; Figure 2c). Net primary production of organic carbon averaged over the harp seal migration area increased with and without anthropogenic nitrogen deposition by +1.08 ± 0.18 and +0.86 ± 0.17 g C m^−2^ decade^−1^, respectively (Figure 2d). Over the 1970 to 2019 period, the anthropogenic nitrogen deposition therefore amplified the increase in net primary production by roughly 4%, closely matching the amplified rate of nitrate delivery due to anthropogenic nitrogen deposition (Figure 2b).

## 4 DISCUSSION

### 4.1 Seals as indicators of environmental change

The Barents Sea harp seal population undertakes annual migrations between their breeding and moulting areas in the southern Barents Sea in late winter and early spring, and Arctic feeding grounds in the Northern Barents Sea in summer and autumn, following the retreating ice northwards (Haug et al., 1994; Nordøy et al., 2008). Teeth GLGs integrate δ^15^N_Phe_ values over one year. Here, we analysed the second and third GLGs of seal individuals that were older than five years. The δ^15^N_Phe_ values used in this study therefore represent a similar timeframe (i.e. 2 years), integrating the δ^15^N_POM_ values at the base of the food web over the entire migration area. As the migration range of this harp seal population is restricted within the Barents Sea, any stressors they experience are a result of environmental forcings occurring in the Barents Sea region. It is worthwhile noting, however, that the annual migration patterns of harp seals depend on the position of the ice edge (Haug et al., 1994; Nordøy et al., 2008), which has retreated north in the past decades (Oziel et al., 2016), potentially affecting the temporal trend in δ^15^N_Phe_ values integrated in their tissues. As the annual mean of δ^15^N_NO3_ values is relatively constant between the Southern Barents Sea (5.0 ± 0.01 ‰) and the Northern Barents Sea (5.1 ± 0.01 ‰; Tuerena et al. (2021)), it is unlikely that a change in migration pattern of harp seals would have a strong effect on the δ^15^N_Phe_ values they integrated during the year. Additional telemetry data from recent years would be needed to confirm that the migration range of harp seals did not vary with time and that the temporal trend in δ^15^N_Phe_ values is not spatially biased.

Overall, the decreasing multi‐decadal change in δ^15^N_Phe_ values in harp seal teeth from the Barents Sea (Figure 1c) agrees well with the overall decline in δ^15^N_POM_ values in the Atlantic sector of the Arctic in our model (Figure 1a) and across most of the harp seal migration area (Figure 1b), albeit at a greater magnitude than simulated, which is discussed further below. The decreasing multi‐decadal trends in both harp seal δ^15^N_Phe_ and simulated δ^15^N_POM_ values in the Barents Sea strongly suggests that the temporal trend in δ^15^N_Phe_ values is mainly driven by environmental change, and not by change in migration pattern. This demonstrates that harp seals from the Barents Sea are good indicators of their environment, and that the multi‐decadal trend in δ^15^N_Phe_ values integrated in their tissue is a reliable record of temporal change in δ^15^N at the base of the food web since the 1950s, extending beyond the time period simulated by the model. Moreover, the reproduction of the multi‐decadal decline in the simulated δ^15^N_POM_ values since the 1970s justifies an exploration of the drivers of this trend using the model.

### 4.2 Drivers of δ^15^N_NO3_ values entering the Barents Sea

**FIGURE 3:**
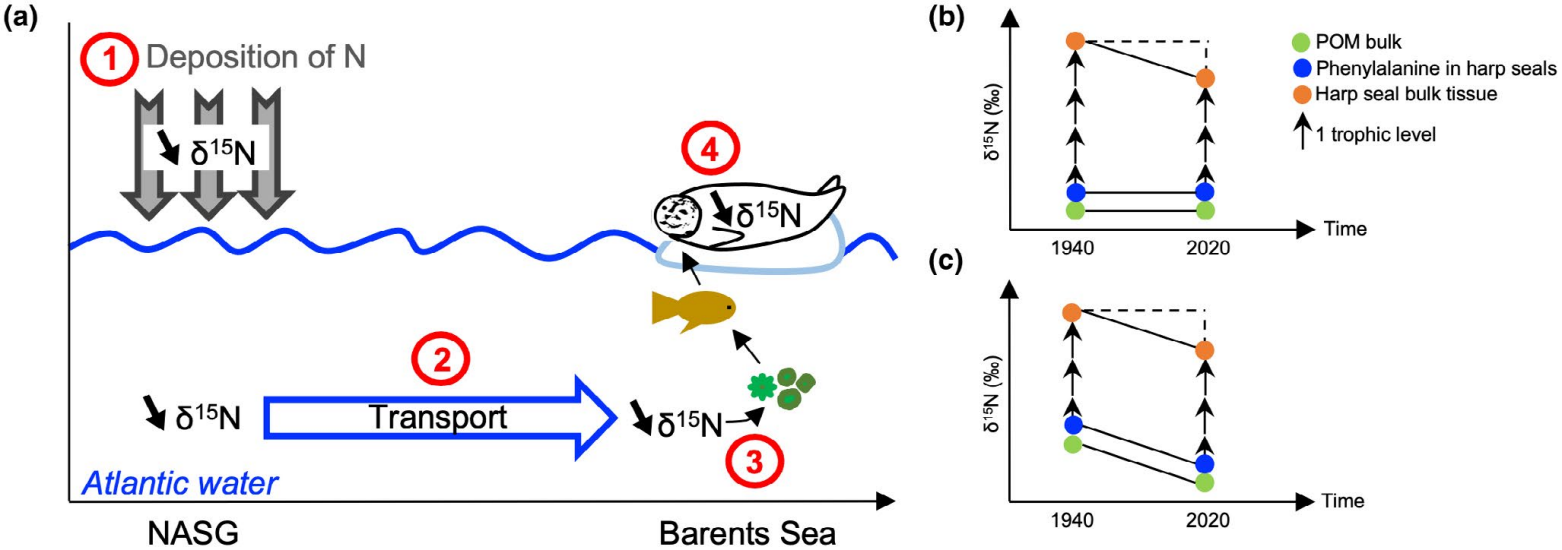
Conceptual schematics of δ^15^N influenced by environmental changes along a food chain in the Barents Sea; (a) Mechanisms driving the multi‐decadal trends in δ^15^N_Phe_ values in harp seals from the Barents Sea: 1 ‐ atmospheric reactive nitrogen deposition (N_r_), 2 ‐ Northward transport of nitrate in Atlantic water, 3 ‐ Nitrate entering the food chain in the Barents Sea, 4 ‐ δ^15^N signature transferred along the food chain to harp seals; NASG = North Atlantic sub‐tropical gyre; (b) Variation in δ^15^N values of bulk tissue in harp seal caused by the loss of one trophic level in the food web; (c) Variation in δ^15^N values of bulk tissue in harp seal caused by variation in the δ^15^N values at the base of the food web

The decline in δ^15^N_NO3_ values at the Barents Sea Opening in both simulations, with and without the anthropogenic increase in nitrogen deposition (Figure 2a), can be explained by several interacting processes. Firstly, Atlantic seawater of subtropical origin is saltier and has a lower δ^15^N_NO3_ signature compared to subpolar seawater due to enhanced evaporation and biological nitrogen fixation in the lower latitudes (Knapp et al., 2008; Marconi et al., 2015). Shallower winter mixing in the subpolar gyre coupled with weakening and westward retraction of the gyre has increased the proportion of subtropical origin water entering the Barents Sea (Hátún et al., 2017), and thus also increased the relative proportion of low δ^15^N_NO3_ values from Atlantic Water. Secondly, the simulated increase in volume transport is consistent with hydrographic observations collected between 1980 and 2011 (Oziel et al., 2016) and satellite‐derived altimetry measurements of current velocities over a 24‐year period (Oziel et al., 2020). A more rapid transport of Atlantic water into the Barents Sea Opening results in an increasingly ^15^N‐depleted δ^15^N_NO3_ endmember, which explains the decrease in δ^15^N_NO3_ values entering the Barents Sea in both simulations. Thirdly, by including the historical invasion of low δ^15^N_NO3_ values to the ocean from atmospheric deposition, we found that the anthropogenic increase in nitrogen deposition was a strong contributor to the low δ^15^N end‐member value of Atlantic water (Figure 3a) and drove the stronger decline in δ^15^N_NO3_ values at the Barents Sea Opening (Figure 2a).

### 4.3 Drivers of δ^15^N_POM_ values within the Barents Sea

Within the Barents Sea, the modelled δ^15^N_POM_ values in the harp seal migration area increased on average without anthropogenic nitrogen deposition, but decreased on average when anthropogenic nitrogen deposition was accounted for (Figure 2c). Meanwhile, and in both simulations, an increasing Atlantic inflow delivered more NO_3_ with lower δ^15^N_NO3_ values to the Barents Sea.

The increase in net primary production in both simulations (Figure 2d) was in accordance with satellite‐derived estimates of chlorophyll, which indicates that net primary production in the Barents Sea has increased since the late 1990s (Dalpadado et al., 2020; Lewis et al., 2020). The increase in net primary production in the Arctic, and especially in the Barents Sea, is primarily due to the decline in sea ice and lengthening of the open water period, but is becoming increasingly controlled by greater nutrient supply (Lewis et al., 2020), particularly nitrate (Tuerena et al., 2021). Increased inflow of warm Atlantic Water (Årthun et al., 2012) is driving a reduction in stratification and an increase in vertical mixing (Lind et al., 2018), which may increase nutrient availability and sustain substantial increases in phytoplankton biomass and production (Lewis et al., 2020; Randelhoff et al., 2020). In our simulations, the flux of nitrate entering the Barents Sea increased (Figure 2b) due to the greater volume of Atlantic origin water (Figure 2b). Despite this influx, nitrate was rapidly used by phytoplankton, resulting in negligible trends in nitrate in the upper 100 m, which is available to phytoplankton, in the Southern Barents Sea and large decrease in the North‐Eastern Barents Sea (Data S2, Figure S2). Because phytoplankton assimilation of nitrate tends to increase both δ^15^N_NO3_ and δ^15^N_POM_ values (Sigman & Fripiat, 2019), the greater nitrate supply (Figure 2b) and associated increase in net primary production (Figure 2d) tended to elevate δ^15^N_POM_ values, despite the increasing delivery of Atlantic Water with low δ^15^N_NO3_ values. The isotopic enrichment associated with increasing primary production therefore overwhelmed the isotopic depletion associated with increasing Atlantic inflow and explained the overall, but slight, increase in mean δ^15^N_POM_ values in simulations without anthropogenic nitrogen deposition (Figure 2c).

However, when the historical increase in atmospheric nitrogen deposition due to anthropogenic activities operated alongside local changes in net primary production and Atlantic inflow, δ^15^N_POM_ values decreased on average. There was a negative decadal trend in δ^15^N_POM_ values over the majority of the Barents Sea in the model (Figure 2c), counterbalancing the trend created by increasing net primary production caused by the additional source of nitrate (Figure 2d). This demonstrates that the historical increase in atmospheric nitrogen deposition, the bulk of which occurred in the second half of the 20th century (Galloway et al., 2004; Yang & Gruber, 2016), was an important driver of the multi‐decadal decline in the δ^15^N_POM_ values. Nonetheless, our modelling suggests that a combination of the anthropogenic nitrogen deposition, greater Atlantic inflow, and a local increase in phytoplankton productivity, all contribute to changes in the δ^15^N_POM_ values at the base of the food web within the Atlantic sector of the Arctic. Furthermore, we demonstrate both observed and simulated declines when all factors are considered, with the δ^15^N_Phe_ values of harp seal teeth revealing declines in the food web baseline since at least the 1950s (Figure 3a).

The stronger decline in δ^15^N_Phe_ values of harp seal teeth compared to simulated δ^15^N_POM_ values could be due to several factors. Although the changes in net primary production (S3‐Fig. 6) and anthropogenic nitrogen deposition are well represented in our model, the isotopic composition of aerosol nitrogen is known to vary from −13‰ to +10‰ and is poorly constrained (Sigman & Fripiat, 2019; Yang & Gruber, 2016). The fixed value of −4‰ attributed to aerosol nitrogen in our simulations may therefore be too high, or fail to represent temporal changes. Second, the relatively coarse model resolution required to simulate global biogeochemical cycling and accurately capture large‐scale nitrogen isotope gradients undoubtedly affected the strength of exchange between the Arctic and its Atlantic neighbours. Mesoscale activity in the region is intense and is essential to replicate realistic rates of Atlantic Water intrusion into the Arctic domain (Fieg et al., 2010; Wekerle et al., 2017). As horizontal grid resolutions less than 10 kilometres are required to resolve the eddy‐driven transport here (Nurser & Bacon, 2014), it is almost certain that our simulated Atlantic Water transport is underestimated. Third, increases in coastal erosion and river runoff in recent decades (Terhaar et al., 2021) were not included in our simulations. However, it is difficult to envision how their inclusion could force a decline in δ^15^N_POM_ values given the significant primary production they support (Terhaar et al., 2021), which would instead increase δ^15^N_POM_ values (Sigman & Fripiat, 2019). Finally, it is difficult to accurately constrain the foraging habitats of the harp seals over the last 60 years, which leads to uncertainties about the appropriate spatial domain over which to compare measured δ^15^N_Phe_ values with simulated δ^15^N_POM_ values. The annual migration patterns of harp seals depend on the position of the ice edge (Haug et al., 1994; Nordøy et al., 2008), which has retreated north in the past decades (Oziel et al., 2016), potentially affecting the temporal trend in δ^15^N_Phe_ values integrated in their tissues. However, this possibility is unlikely due to minor differences in δ^15^N_NO3_ values between the north and south Barents Sea (Tuerena et al., 2021), as discussed earlier. Ultimately, the harp seal and model assessments independently concur in terms of the direction and the multi‐decadal nature of the trends in δ^15^N values at the base of the food web, which persist for 50 years or more in each case.

### 4.4 Consequences of detecting environmental changes in the Arctic ecosystem

Our results demonstrate that the anthropogenic alteration of the Arctic environment may have begun much earlier than previously thought. The invasion of low δ^15^N_NO3_ values into the Barents Sea since the 1950s through a combination of direct deposition of anthropogenic aerosols and increasing inflow of Atlantic seawater clearly demonstrates the sensitivity of the Arctic is to an accumulation of anthropogenic signals. Moreover, it demonstrates how anthropogenic activities, which principally occur at lower latitudes, are communicated to the polar environment.

Alterations to bottom‐up and top down control are known to be critical determinants of marine ecosystem structure and functioning (Boyce et al., 2015). In recent decades, ongoing sea‐ice loss alongside warming of the water column due to Atlantification (Oziel et al., 2016; Polyakov et al., 2020) has driven Arctic species northward and expanded the influence of temperate and boreal species across all trophic levels from phytoplankton (Neukermans et al., 2018; Oziel et al., 2020) to zooplankton (Dalpadado et al., 2016) to fish (Fossheim et al., 2015). Modified trophic interactions have altered the Barents Sea food web with consequences for ecosystem resilience (Pecuchet et al., 2020), which is inherently dependent on the food web structure (Kortsch et al., 2015; Yen et al., 2016). Moreover, increased nutrient availability has raised net primary production, potentially sustaining greater production of consumers (Dalpadado et al., 2020; Lewis et al., 2020; Ware & Thomson, 2005), such as zooplankton (Dalpadado et al., 2020) and fish (Stige et al., 2019). These adjustments to primary production and food web structure will have wider socio‐economic consequences, as the Barents Sea supports some of the world's largest demersal fish stocks important for fisheries, such as Atlantic cod (*Gadus morhua*) and haddock (*Melanogrammus aeglefinus*) (Meredith et al., 2019).

One mechanism by which the changes observed in this study may affect Arctic food webs is through changes in nutrient availability. Nitrate is principally transported into the Arctic via Atlantic inflows across the Barents Sea Opening (Torres‐Valdes et al., 2013). Atlantic waters contain a relative surplus of nitrate relative to other nutrients required by phytoplankton, such as phosphate and silicate, while Arctic surface waters contain a nitrogen deficit (Tremblay et al., 2015). These differences in nutrient availability underpin different phytoplankton communities. The surplus of nitrogen relative to phosphorus and silicate that persists in subtropical Atlantic waters supports a phytoplankton community dominated by smaller, slower growing phytoplankton (Oziel et al., 2020), whereas high phosphorus and silicate is known to favour larger, faster growing phytoplankton, such as diatoms (Moreno & Martiny, 2018). Our results imply an increase in the delivery of nitrate‐rich, but phosphorus‐ and silicate‐poor, waters to the Barents Sea for at least half a century due to a long‐term increase in transport amplified by anthropogenic nitrogen inputs. This is consistent with evidence for growing intrusions of Atlantic water to the region since the early 20th century and altered plankton communities (Tesi et al., 2021). Although the overall phytoplankton community response to altered nutrient ratios is uncertain, evidence for transitions to smaller cells (Li et al., 2009), and away from diatoms is mounting (Ardyna & Arrigo, 2020). As diatoms are selectively grazed by *Calanus* copepods (Meyer‐Harms et al., 1999; Søreide et al., 2008), which are a lipid‐rich keystone species on which higher trophic levels rely (e.g. Falk‐Petersen et al., 2004), a shift away from diatoms due to changes in nutrient availability may propagate up the food web, affecting the productivity of higher trophic levels.

The multi‐decadal decline in δ^15^N_Phe_ values integrating the accumulation of anthropogenic impacts, also has consequences for the study of food webs using stable nitrogen isotopes. Trophic position of top and near‐top predators can be used to assess food‐web structure and is usually determined using δ^15^N values of bulk tissue (δ^15^N_bulk_), which relies on a well constrained baseline for accurate interpretation. Not accounting for changes in values at the base of the food web can lead to a misinterpretation of changes in trophic position of predators (de la Vega et al., 2020), because δ^15^N_bulk_ values varies with both trophic enrichment and δ^15^N_POM_ values that propagates up food webs. The significant decline in δ^15^N_Phe_ values of ~3 ‰ we observed between 1950–1951 and 2015–2016 would represent a change of one trophic level (Post, 2002) assuming a constant baseline (Figure 3b). Instead, we show that this decline reflects a shifting baseline (Figure 3c). Thus, constraining baseline changes and their driving mechanisms is crucial to understand changes in the entire food web and the associated services it provides, such as fisheries, especially in a spatially heterogeneous, rapidly changing environment such as the Arctic.

The range of forcings impacting the Arctic, from local to remote, demands new approaches for monitoring and evaluation that go beyond short‐term, spatially focused, and single disciplinary studies. Our study demonstrates that combining cutting‐edge biomarker techniques, telemetry and state‐of‐the‐art ocean modelling can provide a holistic understanding of how long‐term changes in the Barents Sea arise. The analysis in this paper was underpinned by a unique set of long‐term archive samples, highlighting the importance of long‐term observations in capturing the effects of change. Ultimately, our results suggest that the Barents Sea ecosystem has been impacted by anthropogenically induced changes to the climate and nitrogen cycle for at least 60 years (since the 1950s), extending beyond the time period accessible from satellites and direct oceanographic observations.

## CONFLICT OF INTEREST

The authors declare no conflict of interest.

## Supporting information

Supplementary Material

Supplementary Material

Supplementary Material

## Data Availability

The data that support the findings of this study are openly available in NERC EDS UK Polar Data Centre at https://doi.org/10.5285/6AAA53E8‐3D0A‐48FE‐838E‐31C5B5F24CE7 and in the data base Zenodo at https://doi.org/10.5281/zenodo.6127524.
