# Supplementary material for: Multi‐decadal environmental change in the Barents Sea recorded by seal teeth
Source: Glob Chang Biol. 2022 Mar 1;28(9):3054–65. doi: 10.1111/gcb.16138 (PMC9314922; doi:10.1111/gcb.16138)
Supplement: Supplementary file 1 — Supplementary Material [file GCB-28-3054-s001.docx]

**Supplementary Information 1: harp seal teeth data**

S1-Fig. 1. Schematic illustrating the sampling design for growth layer groups (GLGs); Example of one seal of 5 years old sampled in 1994 and two seals of 8 and 6 years old sampled in 1997.

S1-Fig. 2. (A) residuals *versus* fitted values; the residuals are equally spread around 0: there is minor evidence of heterogeneity. (B) residuals *versus* the explanatory variable “year”; the spread of the residuals is relatively similar at all years: there is minor evidence of non-independence. (C) all the points fall approximately along the reference line: there is minor evidence of non-normality.

S1-Table 1. Parameters of the linear model for δ^15^N_Phe_ of harp seal teeth from the Barents Sea (n = 72); DF = degree of freedom, SD = standard deviation.

| **Response variable** | **Explanatory variable** | **slope** | **SD (slope)** | **p-value (slope)** | **Intercept** | **SD (intercept)** | **p-value (intercept)** | **R^2^ (%)** | **F-statistic (n)** |
| --- | --- | --- | --- | --- | --- | --- | --- | --- | --- |
| δ^15^N_Phe_ | year | -0.036 | 0.005 | < 0.005 | 81.893 | 9.657 | < 0.005 | 43.1 | 54.82 (72) |
