# Supplementary material for: Multi‐decadal environmental change in the Barents Sea recorded by seal teeth
Source: Glob Chang Biol. 2022 Mar 1;28(9):3054–65. doi: 10.1111/gcb.16138 (PMC9314922; doi:10.1111/gcb.16138)
Supplement: Supplementary file 2 — Supplementary Material [file GCB-28-3054-s003.docx]

**Supplementary Information 2: Model results**


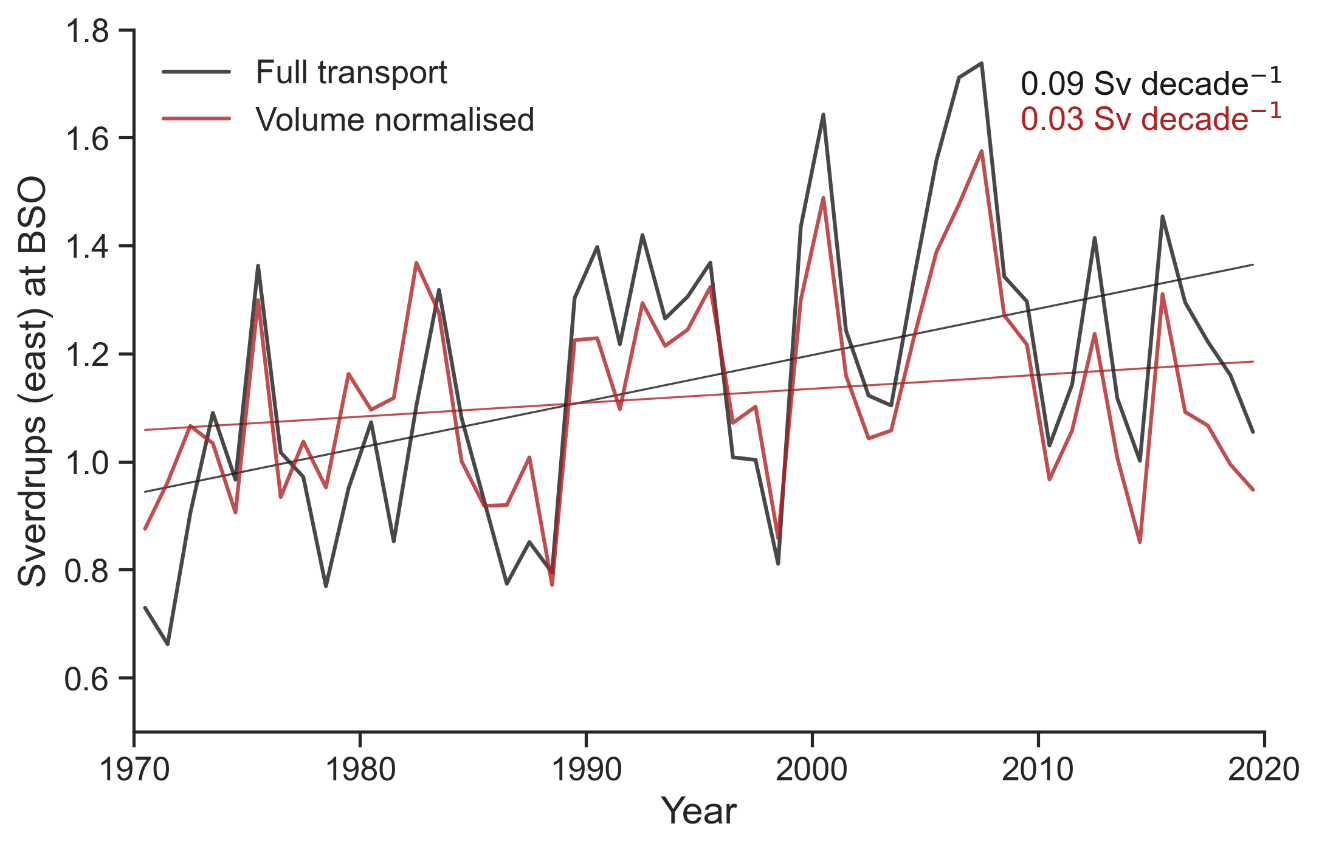


S2-Fig. 1. Simulated transport of Atlantic Water through the Barents Sea Opening (BSO; black) in Sverdrup (Sv = 10^6^ m^3^ s^-1^) and transport normalized by volume of Atlantic Water (red). An increase in the volume of Atlantic Water transported through the Barents Sea Opening is responsible for the difference between the lines and their linear trends.

S2-Fig. 2. Spatiotemporal trends in surface nitrate (NO_3_), (A) decadal trends in NO_3_ averaged over the upper 100 metres of the water column between 1970-2019. The black line indicates the total harp seal migration area and the dark grey line indicates the Barents Sea Opening, (B) inter-annual changes in average upper ocean NO_3_ within the total harp seal migration area.
