# Supplementary material for: Multi‐decadal environmental change in the Barents Sea recorded by seal teeth
Source: Glob Chang Biol. 2022 Mar 1;28(9):3054–65. doi: 10.1111/gcb.16138 (PMC9314922; doi:10.1111/gcb.16138)
Supplement: Supplementary file 3 — Supplementary Material [file GCB-28-3054-s002.docx]

**Supplementary Information 3: model routines and assessment**

Nitrogen isotope routines

Numerical routines of nitrogen isotopes carry the heavy isotopes (^15^N) through nine tracers. These are ammonium (NH_4_), nitrate (NO_3_), both phytoplankton, both zooplankton, large and small particulate organic matter, and dissolved organic matter. The isotopic signature of nitrogen is expressed in delta notation in units of per mil ‰, where:

$$\delta^{15}N=\left( \frac{\left( \frac{\text{15}\text{N}}{\text{14}\text{N}} \right)^{sample}}{\left( \frac{\text{15}\text{N}}{\text{14}\text{N}} \right)^{standard}}-1 \right)\cdot1000$$

$$\left( \frac{\text{15}\text{N}}{\text{14}\text{N}} \right)^{standard}=\left( \frac{\text{15}\text{N}}{\text{14}\text{N}} \right)^{N_{2} in air}= 0.003676$$

Key fractionation processes take place during NO_3_ and NH_4_ assimilation by phytoplankton, denitrification in both the sediments and water column, ingestion of prey by zooplankton, and excretion of NH_4_ by zooplankton. Key sources of ^15^N are atmospheric N_r_ deposition, riverine dissolved inorganic nitrogen, and organic nitrogen fixed by diazotrophs.

All fractionation processes of nitrogen isotopes are biologically mediated, and all follow the same formula. Here, we illustrate the formula of nitrogen isotope fractionation using phytoplankton assimilation of NO_3_ into biomass during primary production.

$${\text{15}N}_{t+1}^{phy}={\text{15}N}_{t}^{phy}+NO_{3}^{assimilated}\cdot\left( \frac{\text{15}\text{N}}{\text{14}\text{N}} \right)^{NO_{3}}\cdot\alpha_{phy}$$

$${\text{15}N}_{t+1}^{NO_{3}}={\text{15}N}_{t}^{NO_{3}}- NO_{3}^{assimilated}\cdot\left( \frac{\text{15}\text{N}}{\text{14}\text{N}} \right)^{NO_{3}}\cdot\alpha_{phy}$$

For each reaction in the nitrogen cycle involving fractionation, the fractionation factor (𝛼) has been experimentally determined by measuring the isotopic ratios of the products and reactants, in this case:

$$\alpha_{phy}=\left( \frac{\left( \frac{\text{15}\text{N}}{\text{14}\text{N}} \right)^{product}}{\left( \frac{\text{15}\text{N}}{\text{14}\text{N}} \right)^{reactant}} \right)=\left( \frac{\left( \frac{\text{15}\text{N}}{\text{14}\text{N}} \right)^{phy}}{\left( \frac{\text{15}\text{N}}{\text{14}\text{N}} \right)^{NO_{3}}} \right)$$

Isotope fractionation factors are close to one and so are often represented in per mil units using the ε notation, where:

$$\varepsilon=\left( 1-\alpha\right)*1000$$

The fraction factors for most reactions are assumed constant, with the exception of NO_3_ and NH_4_ assimilation by phytoplankton. Their default values are:

- Phytoplankton assimilation ($\alpha_{phy}$) = 0.995 ($\varepsilon_{phy}=$5 ‰)
- Water column denitrification ($\alpha_{wc}$) = 0.970 ($\varepsilon_{wc}=$25 ‰)
- Sedimentary denitrification ($\alpha_{sed}$) = 0.997 ($\varepsilon_{sed}= 3$ ‰)
- Nitrification ($\alpha_{nit}$) = 1.0 ($\varepsilon_{nit}=$0 ‰)
- Zooplankton ingestion ($\alpha_{ing}$) = 1.001 ($\varepsilon_{ing}=$-1 ‰)
- Zooplankton excretion ($\alpha_{exc}$) = 0.994 ($\varepsilon_{exc}=$6.0 ‰)

Here, positive ε values indicate an enrichment of ^15^N within the reactant and a depletion in the product.

Fractionation during the assimilation of NO_3_ and NH_4_ by phytoplankton varies according to the ratio of demand and supply of each nutrient. If demand is high but supply is low, such that the amount of nitrogen available to phytoplankton is limiting, then $\alpha_{phy}$ approaches one ($\epsilon_{phy}\to$ 0 ‰). If, however, NO_3_ or NH_4_ are in high concentrations and/or demand is low, then fractionation proceeds at its maximum value, set at $\alpha_{phy}=$0.995 ($\epsilon_{phy}$ = 5.0 ‰). This utilisation effect is calculated by dividing the NO_3_ (NH_4_) required by the NO_3_ (NH_4_) available and is then multiplied against 5.0 ‰.

Sources of ^15^N are atmospheric N_r_ deposition, riverine dissolved inorganic nitrogen, and organic nitrogen fixed by diazotrophs (nitrogen fixers). The signatures of these sources are prescribed using input files during simulations. The default δ^15^N signatures of these sources are as follows:

- Atmospheric N_r_ deposition δ^15^N_DIN_ = -4.0 ‰
- Riverine δ^15^N_DIN_ = 2.0 ‰
- Diazotrophy δ^15^N_org_ = -1.0 ‰

Model assessment: nitrogen stable isotopes of nitrate

We used a global compilation of δ^15^N of nitrate (δ^15^N_NO3_) measurements presented in Rafter et al. (2019), supplemented with data from the Arctic Ocean, to compare with the model. This measurement dataset contained 13096 measurements from all ocean basins over years 1971 to 2018 CE. We made one-to-one comparisons with simulated δ^15^N_NO3_ from the historical simulations by gridding the observations onto the model grid at the appropriate year and month of sampling. If multiple measurements occurred in the same bin, these were averaged. Due to averaging and masking at model land tiles, the model-data comparison involved a total of 8792 comparisons covering approximately 0.8 % of the ocean model volume (S3-Fig. 1).

Simulated δ^15^N_NO3_ provided an adequate overall fit to the *in situ* δ^15^N_NO3_ measurements (S3-Fig. 2). A global correlation of 0.62 and regional correlations ranging from -0.38 to 0.84 revealed the difficulty in reproducing *in situ* δ^15^N_NO3_ measurements. However, these correlations compared well relative to other global ocean models (Buchanan et al. 2019). The worst regional fit, with a correlation of -0.34, was for the Indian Ocean, where the model oxygen field misrepresented the oxygen minimum zones in the Arabian Sea and Bay of Bengal. Because most observations of δ^15^N_NO3_ are from the Arabian Sea, the misplacement of the low oxygen zone had a strong effect on the regional fit. In fact, measurements programs are biased towards sampling within or near to the oxygen minimum zones where denitrification is active, but where the strongest gradients in δ^15^N_NO3_ exist in the modern ocean and where slight biases in model oxygen budgets can have strong effects on nitrogen cycling. The best regional fit, therefore, with a correlation of 0.84, was for the Southern Ocean where the δ^15^N_NO3_ distribution is primarily affected by the physical positioning of water masses and summertime productivity, and the spatial gradients are weaker. All regions suffered from subdued variance compared with the data, which was evident by low normalised standard deviations, but is to be expected when comparing measurements, which are prone to high frequency variability, to monthly-averaged model output.

We assessed the model-data correlation and model bias for each major region and for each year of the simulation (S3-Fig. 3). We accounted for seasonality by ensuring that comparisons were occurring at the correct month. This analysis revealed strong correlations in the Southern Ocean, the absence of a correlation in the Indian Ocean, and more variable correlations in the Atlantic and Pacific. The Arctic Ocean contained one year of poor model-data fit (2014), but returned correlations exceeding 0.6 for other years. Global correlations tended to exceed 0.5, with those lower than 0.5 dragged downwards due to poor agreement in the Indian ocean. All basins contained simulated δ^15^N_NO3_ values that underestimated the measured values, typically by between 0.5 and 1.5 ‰. However, the underestimation appeared consistent across basins, which ensured that the inter-basin differences/gradients of δ^15^N_NO3_ were well reproduced.

Finally, we visually assessed the simulated δ^15^N_NO3_ of the upper 100 metres in the Arctic and compared with data from two cruises, one occurring in July/August of 2017 and the other in May/June of 2018 (S3-Fig. 4). Immediately noticeable is the summertime increase in δ^15^N_NO3_ associated with primary production and nitrogen assimilation by phytoplankton, with higher values in late summer than early summer. The strong regional contrast between the Pacific-influenced and Atlantic-influenced waters is also conspicuous, with Pacific seawater being enriched in ^15^N due to active water column denitrification in this basin and inflow through the Bering Strait of ^15^N-enriched nitrate. Atlantic isotopic signatures are depleted due to active diazotrophy in the North Atlantic, which introduces a low δ^15^N signature to the NO_3_ in this basin. Finally, the model-derived values underestimate the measured values in the upper water column of the Arctic, which as expected are more variable than the monthly-averaged simulated fields. Importantly though, the Atlantic-Pacific gradient is produced. The ~3 ‰ difference between Pacific and Atlantic endmembers in our simulation (S3-Fig. 5) matches observational constraints. Indeed, water column denitrification in the Pacific Ocean leaves an ^15^N-enriched imprint on δ^15^N_NO3_ in the Pacific (Somes et al. 2010), whereas high diazotrophy in the sub-tropical latitudes results in a ^15^N-depleted signature of δ^15^N_NO3_ in the Atlantic (Knapp et al. 2008, Marconi et al. 2015) resulting in a ~3 ‰ difference between Pacific and Atlantic endmembers.

Model assessment: physical state

To determine the performance of the model for simulating surface and circulatory conditions in the Arctic and subarctic North Atlantic, we used observation-based products of sea ice concentration, sea surface temperature (SST) and sea surface height (SSH) to compare with the model output. Observations of sea ice concentration and SST were provided by the National Oceanic and Atmospheric Administration’s Optimal Interpolation SST version 2.1 (OISSTv2.1). The OISSTv2.1 blends remotely sensed daily SST and sea ice concentration from 1981 to present day to produce a global product on a 1/4^th^ degree grid (Reynolds et al. 2007). For SSH, we used output from the Ocean Reanalysis System 4 (ORA-S4) as part of the European Centre for Medium-Range Weather Forecasts (Balmaseda et al. 2013). The ORA-S4 assimilated remotely-sensed and in situ observations of temperature, salinity and sea level anomaly to predict historical ocean conditions from 1959-present.

These datasets were regridded to a 1° by 1° horizontal grid for direct comparison with the model output. Monthly mean sea ice concentrations and SST in the Arctic domain were compared directly using the nonparametric Spearman’s rank correlation, owing to non-normal distributions of these variables. Model-data correlations in sea ice (r=0.97) and SST (r=0.89) were strong, but a seasonal lag was apparent in which the model warmed and cooled too late each year. Meanwhile, SSH was compared in terms of its inter-annual variability. We computed the Subpolar Gyre Index in the North Atlantic (Koul et al. 2020), the North Atlantic Oscillation, and the first empirical orthogonal function in the Arctic domain. We compared the timeseries of these modes of variability between the ORA-S4 and the model using linear correlation (Pearson’s). The model-data comparisons were strong for the Subpolar Gyre Index (r=0.85), the North Atlantic Oscillation (r=0.78), and the principal component timeseries of the first empirical orthogonal function over the Arctic (r=0.85).

Model assessment: chlorophyll-a

8-day composite remotely sensed surface chlorophyll-a concentration from SeaWiFS (1998-2002) and MODIS-Aqua (2003-2018) (processing version R2018) were averaged to a monthly resolution and regridded onto the model domain. This was directly compared to the monthly mean chlorophyll concentrations from 1998-2018 as output by the model. A global spearman’s rank correlation of 0.41 showed moderate agreement. The model tended to underestimate observed chlorophyll, and underestimated both the spatial and temporal variability, as expected due to the model’s coarse resolution. However, increasing trends at the edge of the sea ice, in the Chukchi Sea, Barents Sea and East Siberian Sea were reproduced, as well as the decline in the Kara Sea (S3-Fig. 6).


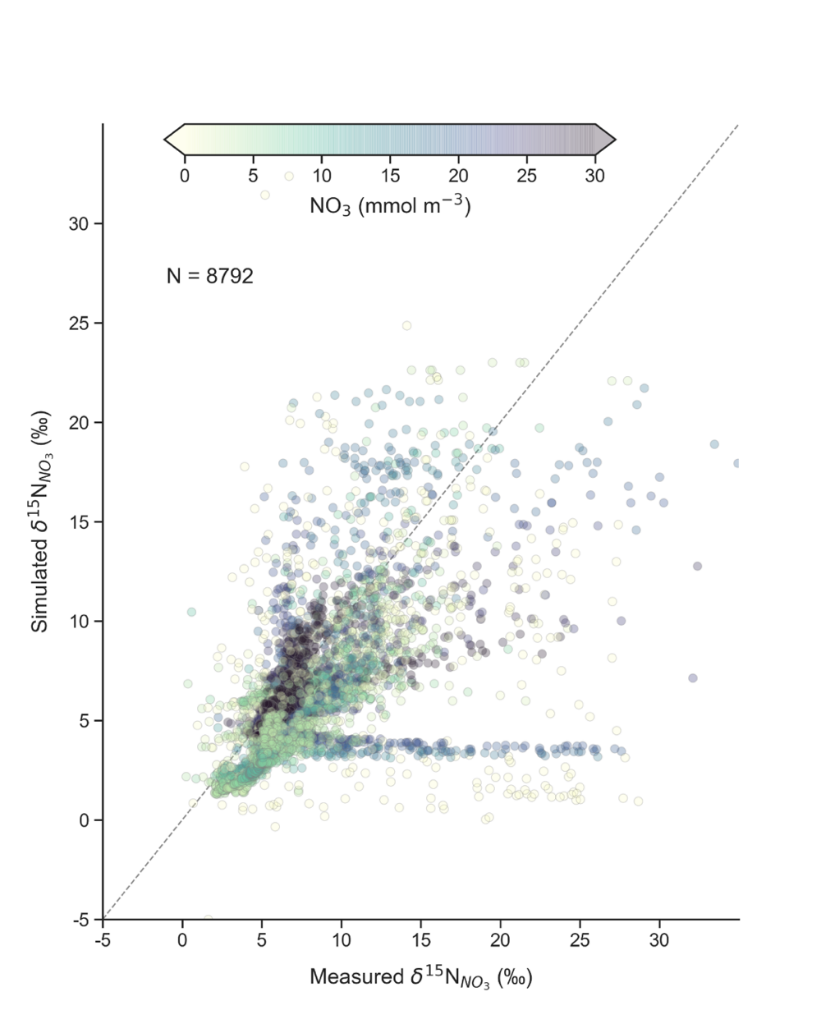


S3-Fig. 1. Direct comparison of measured and modelled δ^15^N_NO3_, coloured by nitrate (NO_3_) concentration.


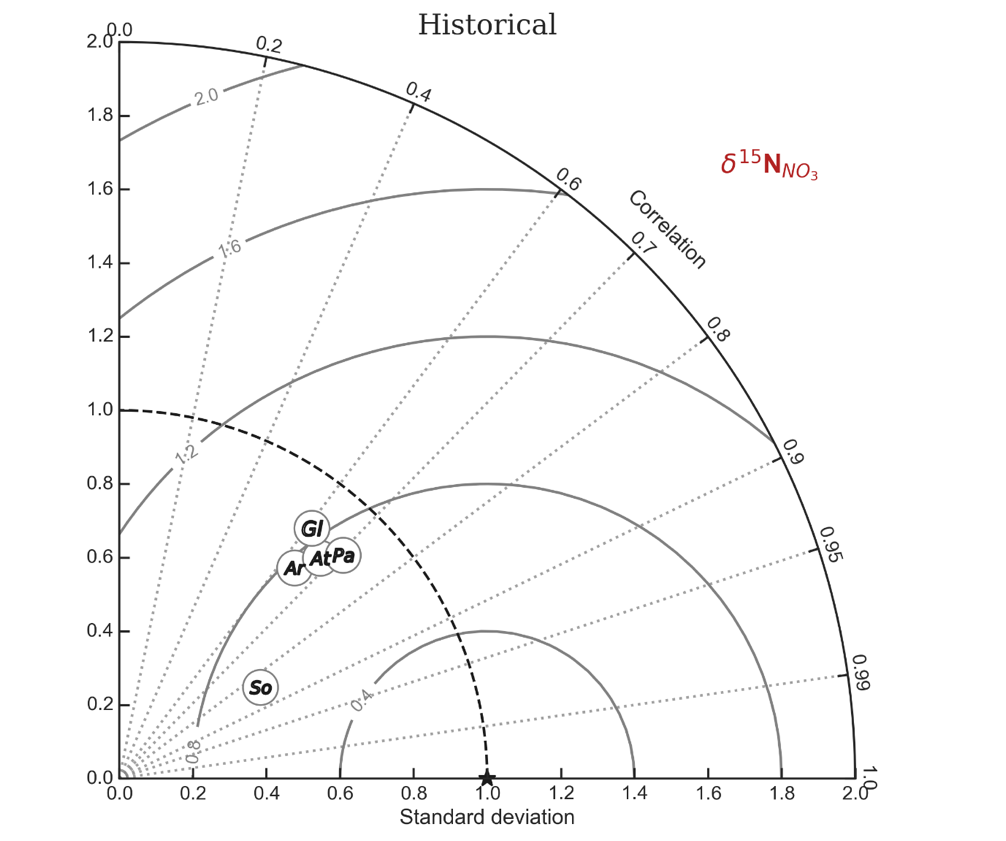
S3-Fig. 2. Taylor diagram (Taylor 2001) summarising the model-data fit for δ^15^N_NO3_. A perfect match between the model and the data would place a marker on top of the star marker, with a correlation of 1.0, a normalised standard deviation of 1.0, and a root mean square error of 0.0. Correlations (Pearson’s r) are represented by radii. Normalised standard deviations are relative to the black dashed line, such that normalised standard deviations less than 1.0 plot below this line. Contours of constant root mean square error are represented by the solid grey lines. Gl = Global; So = Southern Ocean; At = Atlantic; Pa = Pacific; Ar = Arctic. Note that the Indian Ocean does not feature because of a negative correlation.


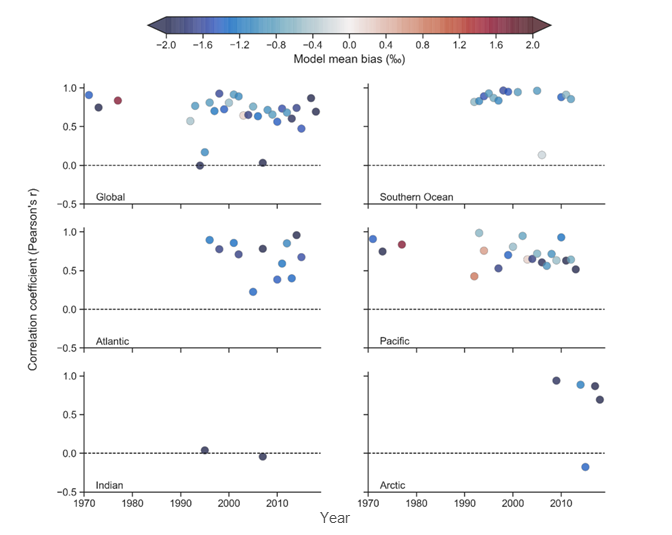


S3-Fig. 3. Correlations and model biases with the global compilation of δ^15^N_NO3_ data for different regions and different years. Comparisons take seasonality into account by comparing observations and model output at the correct month.

S3-Fig. 4. Simulated δ^15^N_NO3_ (shading) and measurements (markers) taken during two cruises in (A) July/August of 2017 and (B) May/June 2018.


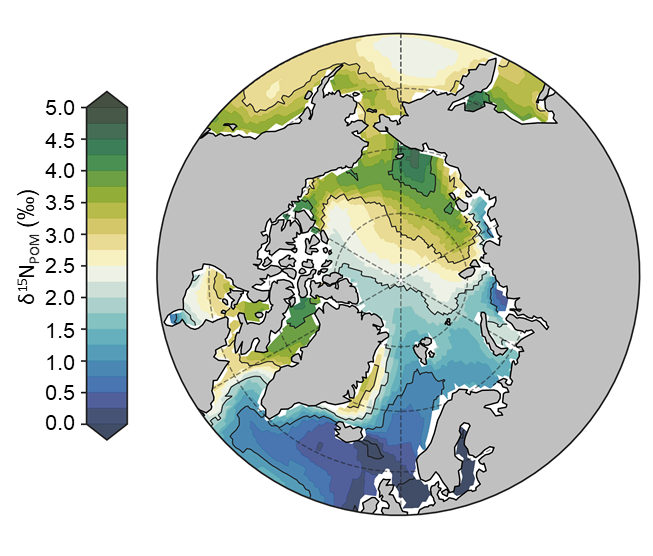


S3-Fig. 5. Spatial variability in average δ^15^N values of particulate organic matter (δ^15^N_POM_) over simulation years 1970-2019 with the historical increase in anthropogenic atmospheric N_r_ deposition.

S3-Fig. 6. (A) Annual trends in remotely sensed chlorophyll-a concentration between 1998-2018 (original 8-day composite data averaged to a monthly resolution and regridded onto the model domain); (B) annual trend in simulated monthly mean surface chlorophyll concentrations from 1998-2018.

S3-References

Balmaseda, M. A., K. Mogensen, and A. T. J. Q. j. o. t. r. m. s. Weaver. 2013. Evaluation of the ECMWF ocean reanalysis system ORAS4. **139**:1132-1161.

Buchanan, P. J., R. J. Matear, Z. Chase, S. J. Phipps, and N. L. Bindoff. 2019. Ocean carbon and nitrogen isotopes in CSIRO Mk3L-COAL version 1.0: a tool for palaeoceanographic research. Geoscientific Model Development **12**:1491-1523.

Knapp, A. N., P. J. DiFiore, C. Deutsch, D. M. Sigman, and F. Lipschultz. 2008. Nitrate isotopic composition between Bermuda and Puerto Rico: Implications for N2 fixation in the Atlantic Ocean. Global Biogeochemical Cycles **22**.

Koul, V., J.-E. Tesdal, M. Bersch, H. Hátún, S. Brune, L. Borchert, H. Haak, C. Schrum, and J. J. S. r. Baehr. 2020. Unraveling the choice of the north Atlantic subpolar gyre index. **10**:1-12.

Marconi, D., M. A. Weigand, P. A. Rafter, M. R. McIlvin, M. Forbes, K. L. Casciotti, and D. M. Sigman. 2015. Nitrate isotope distributions on the US GEOTRACES North Atlantic cross-basin section: Signals of polar nitrate sources and low latitude nitrogen cycling. Marine Chemistry **177**:143-156.

Rafter, P. A., A. Bagnell, D. Marconi, and T. DeVries. 2019. Global trends in marine nitrate N isotopes from observations and a neural network-based climatology. Biogeosciences **16**:2617-2633.

Reynolds, R. W., T. M. Smith, C. Liu, D. B. Chelton, K. S. Casey, and M. G. J. J. o. c. Schlax. 2007. Daily high-resolution-blended analyses for sea surface temperature. **20**:5473-5496.

Somes, C. J., A. Schmittner, E. D. Galbraith, M. F. Lehmann, M. A. Altabet, J. P. Montoya, R. M. Letelier, A. C. Mix, A. Bourbonnais, and M. Eby. 2010. Simulating the global distribution of nitrogen isotopes in the ocean. Global Biogeochemical Cycles **24**.

Taylor, K. E. 2001. Summarizing multiple aspects of model performance in a single diagram. Journal of Geophysical Research: Atmospheres **106**:7183-7192.
